# Transverse Relaxation Time Constant of Cystathionine in Human Glioma at 3 T

**DOI:** 10.1002/mrm.70430

**Published:** 2026-05-20

**Authors:** Dinesh K. Deelchand, Francesca Branzoli, Jamie D. Walls, Lucia Nichelli, Marc Sanson, Bertrand Mathon, Małgorzata Marjańska

**Affiliations:** ^1^ Department of Radiology, Center for Magnetic Resonance Research University of Minnesota Minneapolis Minnesota USA; ^2^ Paris Brain Institute ‐ ICM, INSERM U 1127, CNRS UMR 7225, Sorbonne University, Équipe labelisée LNCC Paris France; ^3^ Department of Chemistry University of Miami Coral Gables Florida USA; ^4^ Sorbonne University, AP‐HP, Paris Brain Institute – ICM, La Pitié Salpêtrière University Hospital ‐ Charles Foix Paris France

**Keywords:** brain, MRS, spectroscopy, T_2_, tumor

## Abstract

**Purpose:**

Cystathionine (Cth) has emerged as a promising biomarker for identifying 1p/19q codeleted gliomas. However, little is known about the T_2_ relaxation time constant of Cth in gliomas. The aim of this study was to measure the T_2_ of Cth in vivo in glioma and compare it to the T_2_s of other metabolites at 3 T.

**Methods:**

Ten participants with glioma were scanned at 3 T. Single‐voxel proton PRESS spectra were acquired at four echo‐times to determine the T_2_ relaxation time constants of Cth, *N*‐acetylaspartate, *scyllo*‐inositol, total creatine, and total choline. Processed spectra were analyzed using LCModel, and the results were fitted using a mono‐exponential function to estimate the T_2_ relaxation time constants. T_2_ of Cth was also measured using high‐resolution NMR.

**Results:**

The T_2_ of Cth varied across participants (42–128 ms, with a mean and standard deviation of 75 ± 24 ms). Additionally, T_2_ relaxation time constants of Cth were shorter than those of singlets measured in glioma in the same participants. Distinct differences in T_2_ between the CH and CH_2_ proton groups in Cth were also observed both in vitro and in vivo.

**Conclusion:**

Knowledge of the T_2_ of Cth should improve its quantification and may help increase understanding of the intracellular environment in glioma cells, potentially providing insights into tumor metabolism in future studies.

## 1 Introduction

Gliomas are a heterogeneous group of primary brain tumors characterized by distinct genetic alterations and metabolic profiles. The integration of metabolic imaging techniques into glioma research and clinical evaluation has enabled non‐invasive probing of tumor biochemistry, offering complementary insights to histopathological and genomic classifications [1, 2]. Cystathionine (Cth) is an emerging metabolic biomarker of interest since recent studies have shown that Cth can help with identifying 1p/19q codeleted gliomas [3, 4], a subtype of isocitrate dehydrogenase (IDH)‐mutated gliomas.

Magnetic resonance spectroscopy (MRS) is uniquely suited for noninvasive detection of Cth in glioma tissue in vivo, which can be achieved using both conventional non‐edited single‐voxel MRS and spectral editing techniques [5, 6]. Interestingly, Cth is undetectable in healthy brain tissue, but appears in significantly higher concentrations in IDH‐mutated gliomas with 1p/19q codeletion compared to IDH‐mutated gliomas lacking this codeletion [4]. This makes Cth a useful indicator for distinguishing these genetic traits within gliomas.

Knowledge of the transverse relaxation time constants (T_2_) of metabolites helps to improve the accuracy of metabolite quantification and provides better insight into how T_E_‐dependent signal decay impacts quantification. In addition, T_2_ provides information about the molecular environment of metabolites, since T_2_ reflects interactions with cellular structures and cytosolic macromolecules. Several studies have reported the T_2_s of metabolites in tumors. For instance, in pediatric brain tumors, the T_2_ relaxation time constants of water and several metabolites such as *N*‐acetylaspartate (NAA) and total choline (tCho) were reported to vary across tissue types [7]. Mixed findings have been reported for the T_2_ of NAA, tCho and total creatine (tCr) in low and/or high‐grade gliomas in adults [8, 9, 10, 11]. Another metabolite of interest in glioma is 2‐hydroxyglutarate (2HG), which accumulates in IDH‐mutated tumors [12, 13]. The T_2_ relaxation time of 2HG was recently reported to be 264 ms in gliomas at 3 T [14].

Despite the growing relevance of Cth metabolism, its T_2_ relaxation property has not yet been reported in vivo. The aim of the current study was therefore to measure the T_2_ relaxation time constant of Cth and compare it with T_2_s of other singlet metabolites in gliomas at 3 T.

## 2 Methods

### 2.1 Participants

Ten participants (8 males, median age: 42 years, range 26–66 years) with gliomas took part in this study after giving informed consent approved by the local ethical committee in accordance with Declaration of Helsinki principles. Additional inclusion criteria were: age > 18 years, Karnofsky performance status > 60, and the ability to provide written informed consent prior to inclusion in the study. The study included 7 low‐grade gliomas (2 astrocytoma grade 2, IDH‐mutated non‐codeleted, and 5 oligodendroglioma grade 2, IDH‐mutated codeleted) and 3 high‐grade gliomas (2 oligodendroglioma grade 3, IDH‐mutated codeleted, and 1 glioblastoma, IDH‐wild‐type). Eight participants were scanned before treatment, including surgery; one participant with astrocytoma grade 2 had a history of previous treatment and was scanned 2 weeks after beginning of chemotherapy; and one participant with oligodendroglioma grade 2 was scanned 2 weeks after beginning of ivosidenib treatment. All scans were carried out on the 3 T Siemens Prisma scanner (Siemens, Erlangen, Germany). A standard body coil was used for excitation while a 64‐channel receive‐only head‐coil was used for signal reception.

### 2.2 MR Acquisitions

3D FLAIR T_2_ images (FOV = 256 × 256 × 176 mm^3^, resolution: 1.0 × 1.0 × 1.0 mm^3^, T_R_/T_E_ = 8000/393 ms) were acquired to guide accurate placement of the MRS volume‐of‐interest (VOI). The VOI size was adapted to the tumor size, resulting in a mean ± SD volume of 9.8 ± 1.9 mL (range 7.2–13.2 mL) across all participants.

FAST(EST)MAP [15] was used to optimize the 1^st^ and 2^nd^ order shims within the MRS VOI, followed by VOI‐based calibrations of water suppression pulses. Single‐voxel MRS data were acquired using a PRESS sequence [16] which consisted of a 2.32 ms Hanning‐windowed sinc excitation pulse (bandwidth = 4.67 kHz) and 5.8 ms Mao refocusing pulses (bandwidth = 1.3 kHz). Water suppression and elimination of extraneous signals were achieved by interleaving VAPOR with outer‐volume suppression pulses [17]. MRS data (T_R_ = 2 s, 2048 complex points and spectral bandwidth = 3 kHz) were acquired at four echo‐times (T_E1_/T_E2_): 23.64 (12/11.64), 68 (13/55), 97 (32/65), and 110 (30/80) ms with 64 transients. With this version of the PRESS sequence, the minimal achievable T_E_ was 23.64 ms. The T_E1_ = 13 ms for the T_E_ = 68 ms acquisition was based on the minimum delay between the excitation pulse and the 1^st^ refocusing pulse. The inter‐pulse delays for T_E_ = 97 ms were adapted from Choi et al. [13] where the timing was optimized to detect 2HG. At T_E_ of 110 ms, T_E1_ and T_E2_ were optimized using density matrix simulations to better detect the Cth signal while minimizing the overlap with the aspartate (Asp) signal (Figure S1). The PRESS data at T_E_ = 68 ms were acquired using MEGA‐PRESS sequence in the edit‐off condition (editing pulse at 7.5 ppm). Water reference scans were also collected for eddy current correction with the RF pulses disabled in the VAPOR module, using the same T_E_s and T_R_ as described above.

Single‐voxel MEGA‐PRESS data [18] (T_R_/T_E_ = 2000/68 ms, 128 pairs of transients) were also acquired in all participants. The editing pulse (19.2 ms SLR pulse with 62 Hz bandwidth) was applied in an interleaved fashion at 1.9 ppm for edit‐on condition and at 7.5 ppm for edit‐off condition.

### 2.3 MRS Analysis

All single‐shot MRS data were pre‐processed in MATLAB (The MathWorks, Massachusetts, USA) with eddy‐current, frequency and phase corrections. Summed spectra were then analyzed with LCModel [19] using simulated PRESS basis sets (1 Hz linewidth) at the three measured T_E_s (23, 97 and 110 ms). For T_E_ = 68 ms, the MEGA‐PRESS sequence was simulated with the edit‐off condition, where the editing pulse was placed at 7.5 ppm. During density‐matrix simulation of the basis spectra, the RF pulse profiles, inter‐pulse delays and the localization of the refocusing pulses were incorporated as previously described [16]. All PRESS basis sets included the following 25 metabolites: 2HG, alanine, ascorbate, Asp, betaine, creatine (Cr), citrate, Cth, γ‐aminobutyric acid, glucose, glutamate, glutamine, glutathione, glycerophosphorylcholine, glycine, *myo*‐inositol, *scyllo*‐inositol (sIns), lactate, NAA, *N*‐acetylaspartylglutamate (NAAG), phosphocreatine (PCr), phosphorylcholine, phosphorylethanolamine, serine and taurine. Cr and PCr were each divided into two basis spectra corresponding to the methylene (CH_2_) and methyl (CH_3_) resonances. Similarly, NAA was represented by two basis spectra, singlet and multiplet NAA. Note that Cth basis was simulated as a single spectrum, assuming all proton groups have identical T_2_ relaxation time constants. The measured macromolecule spectra from prior studies of healthy participants [20, 21] were included in the analysis only for T_E_s of 23.64 ms and 68 ms since their contributions at longer T_E_ were minimal. The edited MEGA‐PRESS basis set contained a measured macromolecule spectrum and simulated 2HG, Cth, γ‐aminobutyric acid, glutamate, glutamine, glutathione, NAA and *N*‐acetylaspartylglutamate basis spectra with a 1 Hz linewidth. The LCModel fitting range was from 0.5 to 4.1 ppm for the non‐edited data and from 1.8 to 4.2 ppm for the edited data, with a spline knot spacing of 5.

The LCModel reported amplitudes of Cth (entire molecule), and the singlet resonances (sIns, tCr‐CH_3_ (i.e., the methyl group of Cr + PCr at 3.03 ppm), tNAA (singlet NAA + NAAG) and tCho) were fitted using a single‐exponential model in MATLAB to estimate the T_2_ values of these metabolites. Goodness of fit was assessed using R^2^. A Pearson correlation analysis was performed to assess the relationship between the T_2_ relaxation time constants of Cth and those of the other metabolites. The reported SNR was measured in the frequency domain and defined as the peak height of tCr at 3.03 ppm divided by root mean square of the noise measured between −2 and 0 ppm. More details are provided in Table S1.

Correlations between T_2_ of Cth and T_2_ of other metabolites (tNAA, tCr‐CH_3_) were assessed using Pearson correlation coefficients. Statistical significance was evaluated with two‐tailed *p*‐values, with significance set at *p* < 0.05.

### 2.4 High‐Resolution NMR

5 mg of L‐Cth (≥ 98% purity, item #16061) was purchased from Cayman Chemical (Ann Arbor, MI, USA) and dissolved in D_2_O to prepare a 300 μL 75 mM solution. High‐resolution NMR experiments were performed on a Bruker 600 MHz vertical‐bore spectrometer using the PROJECT pulse sequence [22]. Acquisition parameters included a repetition time of 10 s, 16 transients, a spectral bandwidth of 5 kHz, and 32 K complex points. The inter‐pulse delay was set to 1.2 ms. To determine the T_2_ relaxation time constants of individual proton groups, 13 time points were acquired using varying numbers of pulse train repetitions which resulted in total echo times ranging from 91 to 1645 ms. The peak amplitudes for each proton group were fitted using a single‐exponential function in MATLAB to estimate the T_2_ values.

### 2.5 T_2_ ‐Corrected Cth Basis Spectrum

To account for T_2_ effects at T_E_ = 68 ms, the methine (CH) and CH_2_ groups of Cth were simulated separately. Each component was adjusted using the T_2_ values obtained from the high‐resolution NMR measurements by modifying its spectral linewidth and correcting its signal amplitude for T_E_ = 68 ms in both the PRESS and the edited MEGA‐PRESS basis spectra of Cth. The CH and CH_2_ signals were then combined to generate a single Cth basis spectrum. This modified basis spectrum is referred to as the T_2_‐corrected basis spectrum, whereas the original Cth basis spectrum is referred to as the standard basis spectrum.

## 3 Results

**FIGURE 1:**
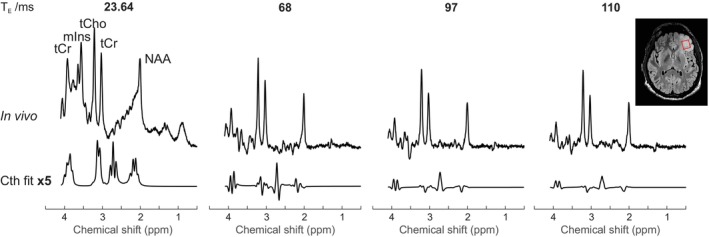
^1^H PRESS spectra acquired at four echo times in a human participant with IDH‐mutated, non‐codeleted glioma at 3 T. The measured spectra and the LCModel‐fitted Cth spectra, with amplitudes scaled by a factor of 5, are shown. The insert shows the VOI location on the anatomical image.

Figure 1 shows the PRESS spectra from one participant acquired at four echo times, along with the corresponding fits of Cth. The LCModel‐fitted Cth spectra clearly show a decrease in peak amplitude for the multiplet around 2.7 ppm. As expected, changes in the peak amplitudes of singlets such as tNAA, tCr, and tCho are also noticeable in the figure. The spectral SNR across all data was excellent; at the longest T_E_ of 110 ms, the measured SNR of tCr‐CH_3_ was 59 ± 16. The reported LCModel linewidth for each participant across T_E_s is reported in Table S2.

No significant correlation was found between Asp and Cth at the shortest T_E_ of 23 ms (r ranging from −0.36 to −0.07 across participants). Although Asp was fitted at 23 ms, it was associated with a higher CRLB compared to Cth (Figure S1, Table S3). At longer T_E_ (≥ 68 ms), the reliability to quantify Asp was much lower compared to Cth. The Cth signal was relatively high in all participants. Across T_E_s, no correlation was observed between Cth and other metabolites resonating at 2.14 ppm and 3.074 ppm, such as tCr‐CH_3_, singlet NAA, and NAAG.

**FIGURE 2:**
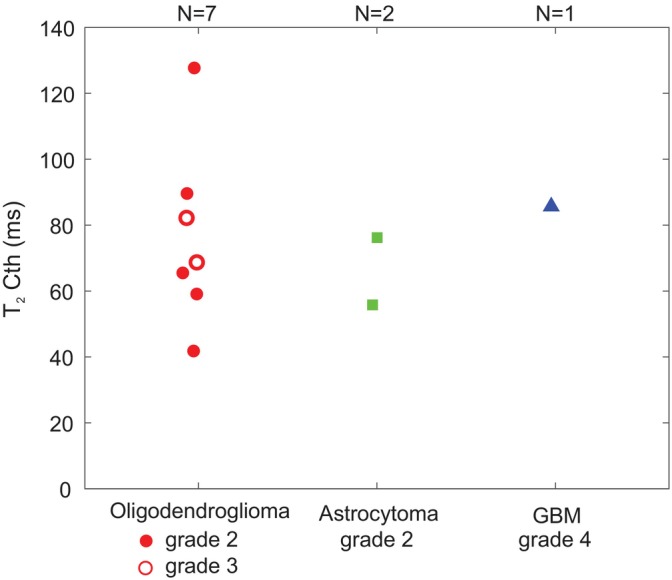
Cth T_2_ values (in ms) in different glioma types. Each point represents one participant, with colors indicating oligodendroglioma (red), astrocytoma (green), and glioblastoma (GBM) (blue).

The T_2_ relaxation time constants of Cth were measured in all 10 participants and exhibited considerable inter‐subject variability, ranging from 48 to 128 ms across the study cohort, with a mean ± SD T_2_ of 75 ± 24 ms (mean R^2^ > 0.98, Table 1, Figure S2). This measured T_2_ of Cth represents the relaxation of the entire molecule (i.e., both CH and CH_2_ groups), as the LCModel fit relies on the CH_2_ resonance at ˜2.7 ppm, where spectral overlap is minimal. Comparable variability was observed for sIns, tNAA, tCr‐CH_3_, tCho and water, as reflected by their standard deviations (Table 1). A weak negative correlation trend was observed between the T_2_ of Cth and tNAA (*r* = −0.396, *p* = 0.27), and a weak positive correlation trend between the T_2_ of Cth and tCr‐CH_3_ (*r* = 0.28, *p* = 0.43), though neither relationship reached statistical significance. Figure 2 shows the distribution of Cth T_2_ values across participants for each of the three tumor types included in this study. The average T_2_ of Cth in grade 2 tumors was 74 ± 28 ms (*N* = 7), 75 ± 10 ms in grade 3 tumors (*N* = 2), and 86 ms in grade 4 tumor (*N* = 1).

**TABLE 1:** T_2_ relaxation time constants (mean ± SD and range) of different metabolites in glioma tissue measured in 10 participants with glioma at 3 T.

	Mean ± SD T_2_ (ms)	Range of T_2_ values (ms)
Cth (entire molecule)	75 ± 24	42–128
sIns	100 ± 43	46–158
tCho	238 ± 47	182–340
tCr‐CH_3_	154 ± 14	138–174
tNAA	310 ± 117	113–474
Water	146 ± 33	111–201

*Note*: tNAA represents the sum of the NAA singlet and NAAG signals.

**FIGURE 3:**
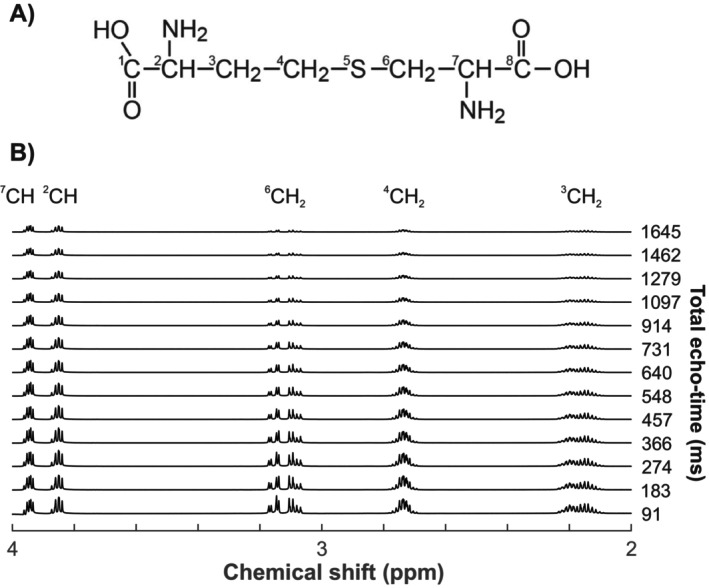
(A) Structure of Cth. (B) High‐resolution ^1^H NMR spectra of Cth acquired at different echo times using the PROJECT [22] sequence (T_R_ = 10 s, inter‐pulse delay of 1.2 ms, 16 transients per spectrum, 37°C) on a 600 MHz spectrometer. Protons from the different Cth groups are indicated above. The CH groups have longer T_2_ relaxation time constants than the CH_2_ groups, as signals from the CH groups are still visible at the longest echo time of 1645 ms, while signals from the CH_2_ groups are almost invisible.

Figure 3 shows high‐resolution NMR spectra of Cth acquired using the PROJECT sequence with various echo times. As expected, no *J*‐modulation was observed for this *J*‐coupled metabolite. The signals from all methylene group protons were nearly absent at the longest T_E_ of 1645 ms, in contrast to the more persistent signals from methine group protons. The measured T_2_ relaxation time constants for the three CH_2_ groups were comparable, with a mean ± SD value of 750 ± 35 ms, while the two CH groups exhibited a longer mean ± SD T_2_ of 1662 ± 103 ms.

To take into account the difference in T_2_ of different groups of Cth protons, the T_2_‐corrected Cth basis spectrum was simulated at TE = 68 ms. The amplitude of CH_2_ groups was scaled down relative to CH groups, assuming their T_2_ is 2.2 times shorter, and the T_2_ of CH_2_ groups is 75 ms. Additionally, a spectral linewidth of 0.45 Hz (i.e., narrower due to longer T_2_ values) was used for the CH groups, while the linewidth of the CH_2_ groups was kept at 1 Hz. Edited and edit‐off PRESS spectra at TE = 68 ms were fitted using the standard (assuming identical T_2_ values for all Cth proton groups) and T_2_‐corrected Cth basis spectra.

**FIGURE 4:**
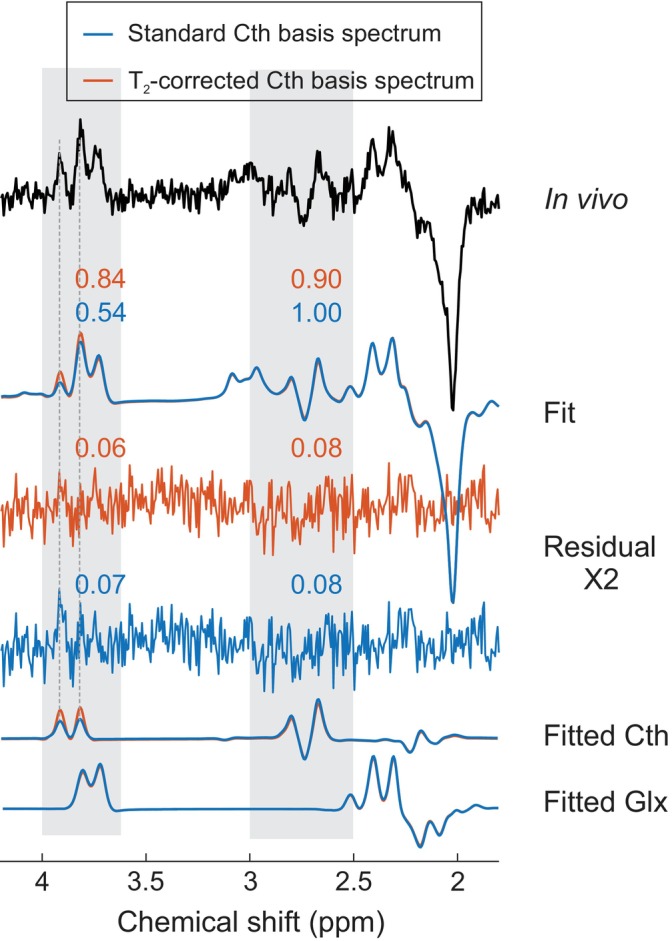
Effect of using standard (blue) and T_2_‐corrected (red) Cth basis spectra to fit the edited MEGA‐PRESS spectrum (T_E_ = 68 ms, 128 pairs of transients) from one participant (IDH‐mutated codeleted) with high Cth level. Smaller residuals and higher Cth amplitudes in the 3.85 ppm region (dotted vertical lines) were observed when using the T_2_‐corrected Cth basis spectrum. The reported numbers represent the normalized amplitude of Cth (above the fits) and the RMS of the residuals in two spectral regions (gray bands). The difference in glutamate + glutamine (Glx) concentration was small when using different basis sets.

Figure 4 shows the LCModel fits of edited data acquired at T_E_ = 68 ms in a single participant with the highest Cth concentration. This participant's data is shown to illustrate the fact that Cth groups have different T_2_ values in vivo. When the standard Cth basis spectrum was used, residuals were present around the CH region (˜3.85 ppm), but not around 2.7 ppm. The residuals were more apparent in the edited data than in the PRESS data (not shown) due to fewer overlapping metabolites near 3.85 ppm. In contrast, when the basis spectrum was modified to reflect the experimentally determined T_2_ differences between the CH and CH_2_ groups (based on the high‐resolution data), the residuals were less pronounced and the fit of the CH group improved markedly. The fitted amplitudes for the Cth‐CH resonances were higher when the T_2_‐corrected basis spectrum was used in both datasets. Glutamate + glutamine concentration was slightly reduced (˜5%) when using the T_2_‐corrected Cth basis set.

## 4 Discussion

This study reports the T_2_ relaxation time constants of Cth in participants with glioma, finding them to be short, with a mean ± SD of 75 ± 24 ms. The measured T_2_ of Cth varied across participants, likely reflecting differences in the intracellular environment of glioma cells and/or metabolic composition, and is potentially influenced by the subtype and stage of tumor growth [6]. To our knowledge, this is the first study reporting the T_2_ relaxation properties of Cth both in vitro and in vivo.

In this study, we found that the CH and CH_2_ groups in Cth have different T_2_ relaxation time constants both in vitro (Figure 3) and in vivo (Figure 4). Typically, differences in T_2_ have been reported between non‐coupled groups within the same molecule in vivo [20, 23, 24]. For example, the T_2_ of the tCr‐CH_3_ is longer than the T_2_ of the tCr‐CH_2_ group in the human brain. Similarly, in NAA, the T_2_ values of the singlet (CH_3_) and multiplet (CH_2_) protons differ. In vitro, it is also common to observe different T_2_ between *J*‐coupled and non‐coupled protons within the same molecule [25, 26]. Therefore, the observation that CH and CH_2_ protons in Cth exhibit distinct T_2_ values in vivo is somewhat surprising, given that the ^2^CH proton is coupled to the ^3^CH_2_ protons, and the ^7^CH proton is coupled to the ^6^CH_2_ protons. This difference likely arises from the influence of the sulfur bridge in the thioether moiety which creates unique local chemical environments and molecular dynamics. Interestingly, the T_2_ ratio between the CH and CH_2_ groups in Cth appears to be independent of magnetic field strength. This is most likely due to the dominant intramolecular dipole–dipole interactions, which exhibit a weak B_0_ dependence.

The mean T_2_ relaxation time constants of tNAA, tCr‐CH_3_, and tCho measured in glioma tissues in the current study, which includes a mixture of low and high‐grade tumors, were comparable to previously reported values in white matter tissue in the healthy brain at 3 T [8, 9, 24, 27, 28, 29], which are consistent with findings from other brain regions [11, 20, 24, 30]. In these studies, the reported T_2_ ranges were: NAA, 240–309 ms; tCr‐CH_3_, 139–175 ms; and tCho, 169–319 ms. In contrast, studies in glioblastoma have reported variable results: three studies reported a longer T_2_ for tCho [8, 9, 10], while one study found a shorter T_2_ [11]. Similar observations were reported for the T_2_ of tCr‐CH_3_ in glioblastoma. For NAA in glioblastoma, two studies reported shorter T_2_ compared to controls [10, 11] while others reported no difference [8, 9]. These mixed results across studies can be likely attributed to differences in tumor grade or tissue composition [31]. In the current study, the measured T_2_ of water in glioma was higher compared to healthy brain tissue [20], consistent with previous studies [7, 10]. Remarkably, the T_2_ of sIns was shorter in gliomas than in healthy brain [32], indicating that metabolite relaxation is influenced by microenvironmental factors distinct from those affecting bulk water.

In summary, the T_2_ relaxation time constants of Cth were successfully measured in participants with glioma. The observed variation across participants is most likely related to intracellular differences in tissue types. Additionally, the CH and CH_2_ groups in Cth have different T_2_s both in vitro and in vivo. Furthermore, the T_2_ of Cth was shorter than that of singlets in glioma. By reporting the T_2_ of Cth in gliomas at 3 T, this study provides fundamental information that may help improve Cth quantification and understanding of the intracellular environment and tumor metabolism in future research.

## Funding

This work was supported by Agence Nationale de la Recherche, ANR‐20‐CE17‐0002‐01. National Institutes of Health, P41EB015894, R01EB034231, U01CA269110. Investissements d'avenir, ANR‐10‐IAIHU‐06, ANR‐11‐INBS‐0006.

## Supporting information


**Figure S1:** Simulated spectra of Cth (red) and Asp (blue) at four echo times for two conditions: (A) identical concentrations and (B) different concentrations with in vivo observed linewidth of 6 Hz. The spectral overlap between the two metabolites is not substantial, suggesting that Asp does not strongly confound the quantification of Cth under either condition at these echo times.
**Figure S2:** Exponential T_2_ fits from three participants representing short, intermediate and long T_2_ values of Cth. The corresponding T_2_ values and R^2^ (goodness‐of‐fit) are listed in the table on the right.

**Table S1:** Minimum reporting standards in the MR spectroscopy checklist.
**Table S2:** FWHM values (in Hz) reported by LCModel across T_E_s and participants.
**Table S3:** Mean ± SD and range of CRLB (%) across T_E_s for Asp and Cth for all participants.

## Data Availability

The data that support the findings of this study are available on request from the corresponding author. The data are not publicly available due to privacy or ethical restrictions.
