# Supplementary material for: Transverse Relaxation Time Constant of Cystathionine in Human Glioma at 3 T
Source: Magn Reson Med. 2026 May 20;96(3):1076–82. doi: 10.1002/mrm.70430 (PMC13327420; doi:10.1002/mrm.70430)
Supplement: Supplementary file 1 — Figure S1: Simulated spectra of Cth (red) and Asp (blue) at four echo times for two conditions: (A) identical concentrations and (B) different concentrations with in vivo observed linewidth of 6 Hz. The spectral overlap between the two metabolites is not substantial, suggesting that Asp does not strongly confound the quantification of Cth under either condition at these echo times. Figure S2: Exponential T2 fits from three participants representing short, intermediate and long T2 values of Cth. The corresponding T2 values and R2 (goodness‐of‐fit) are listed in the table on the right. [file MRM-96-1076-s001.doc]

**
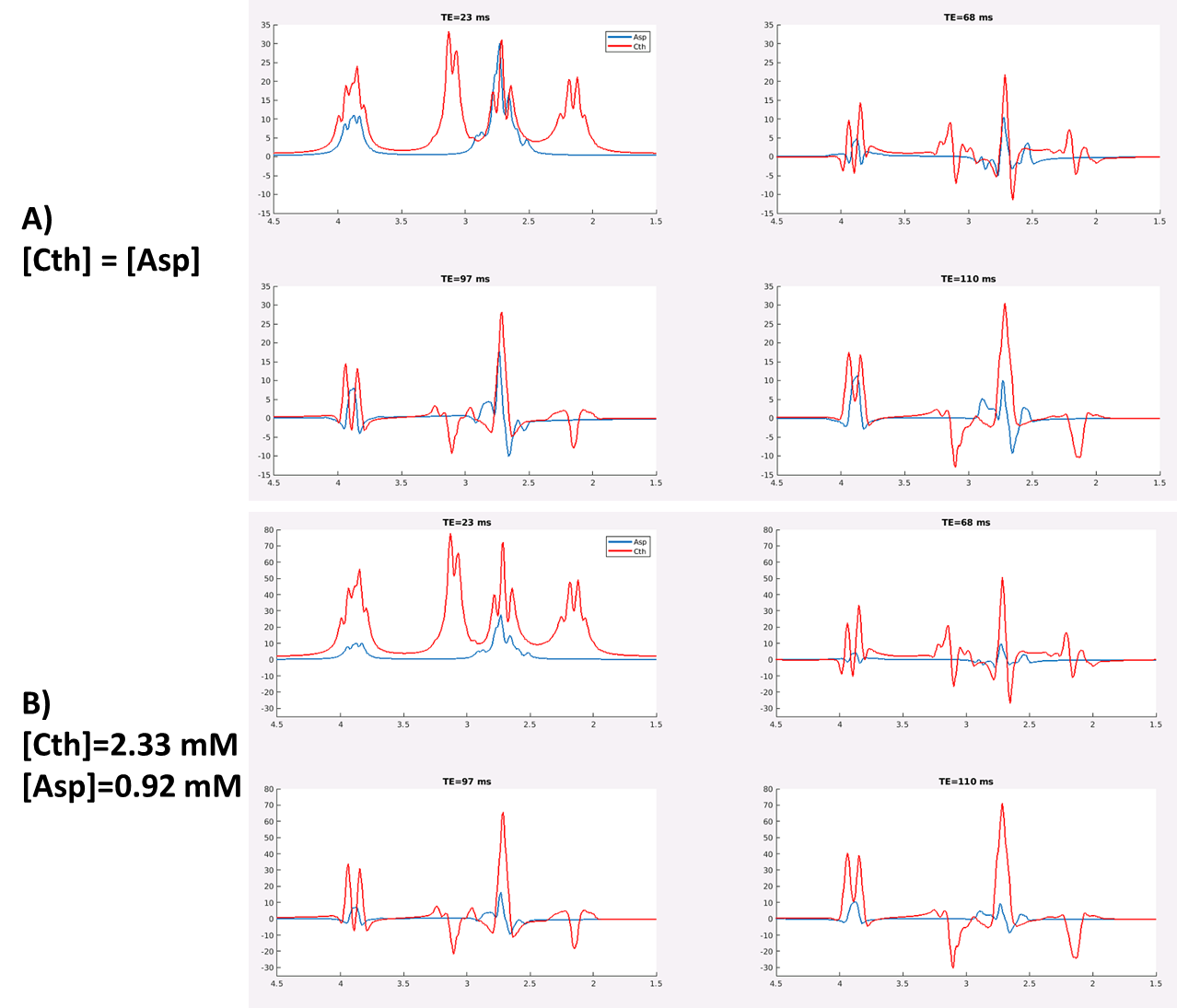
**

**Figure S1**: Simulated spectra of Cth (red) and Asp (blue) at four echo times for two conditions: (A) identical concentrations and (B) different concentrations with *in vivo* observed linewidth of 6 Hz**.** The spectral overlap between the two metabolites is not substantial, suggesting that Asp does not strongly confound the quantification of Cth under either condition at these echo times.

**
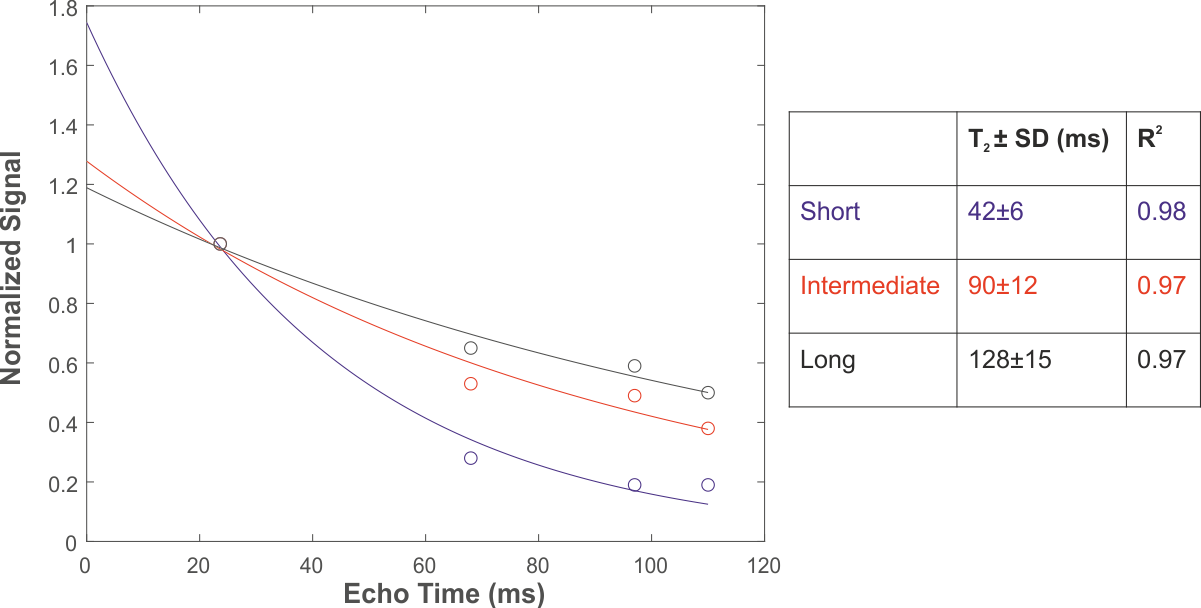
**

**Figure S2**: Exponential T_2_ fits from three participants representing short, intermediate and long T_2_ values of Cth. The corresponding T_2_ values and R^2^ (goodness-of-fit) are listed in the table on the right.
