# Supplementary material for: Transverse Relaxation Time Constant of Cystathionine in Human Glioma at 3 T
Source: Magn Reson Med. 2026 May 20;96(3):1076–82. doi: 10.1002/mrm.70430 (PMC13327420; doi:10.1002/mrm.70430)
Supplement: Supplementary file 2 — Table S1: Minimum reporting standards in the MR spectroscopy checklist. Table S2: FWHM values (in Hz) reported by LCModel across TEs and participants. Table S3: Mean ± SD and range of CRLB (%) across TEs for Asp and Cth for all participants. [file MRM-96-1076-s002.doc]

**Table S1**. Minimum reporting standards in the MR spectroscopy checklist

| 1. Hardware |  |
| --- | --- |
| a. Field strength [T] | 3 T |
| b. Manufacturer | Siemens |
| c. Model (software version if available) | Prisma (Syngo VE11C) |
| d. RF coils: nuclei (transmit/receive), number of channels, type, body part | Transmit: body coil  Receive: 64-channel head coil |
| e. Additional hardware | N/A |
| 2. Acquisition |  |
| a. Pulse sequence | CMRR developed PRESS and MEGA-PRESS |
| b. Volume of Interest (VOI) locations | Tumor region |
| c. Nominal VOI size [cm^3^, mm^3^] | 7.2 – 13.2 mL |
| d. Repetition Time (TR), Echo Time (TE) [ms, s] | TE=23.64 ms, TE1/TE2 = 12/11.64 ms  TE=68 ms, TE1/TE2 = 13/55 ms  TE=97 ms, TE1/TE2 = 32/65 ms  TE=110 ms, TE1/TE2 = 30/80 ms  TR = 2000 ms |
| e. Total number of Excitations or acquisitions per spectrum  In time series for kinetic studies   1. Number of Averaged spectra (NA) per time-point 2. Averaging method (e.g. block-wise or moving average) 3. Total number of spectra (acquired / in time-series) | 64 and 128 transients for PRESS and edited data, respectively  Stored as individual FIDs |
| f. Additional sequence parameters (spectral width in Hz, number of spectral points, frequency offsets)  If STEAM:, Mixing Time (TM)  If MRSI: 2D or 3D, FOV in all directions, matrix size, acceleration factors, sampling method | Spectral width = 3 kHz  2048 complex points  Frequency offset = 3 ppm |
| g. Water Suppression Method | VAPOR with suppression bandwidth of 68 Hz |
| h. Shimming Method, reference peak, and thresholds for “acceptance of shim” chosen | FAST(EST)MAP B0 shimming |
| i. Triggering or motion correction method  (respiratory, peripheral, cardiac triggering, incl. device used and delays) | N/A |
| 3. Data analysis methods and outputs |  |
| a. Analysis software | In-house matlab scripts |
| b. Processing steps deviating from quoted reference or product | Single-shot frequency and phase corrections followed by eddy current correction |
| c. Output measure  (e.g. absolute concentration, institutional units, ratio) | Metabolite amplitudes were quantified using LCModel v6.3-0G |
| d. Quantification references and assumptions, fitting model assumptions | Few LCModel “CONTROL” parameters:  DKNTMN=5  NSIMUL=6 where Lip13a, Lip13b, Lip13c, Lip13d, Lip09 and MM09 were included during the fit |
| 4. Data Quality |  |
| a. Reported variables  (SNR, Linewidth (with reference peaks)) | SNR of tCr = 59 at TE=110ms |
| b. Data exclusion criteria | No subjects excluded |
| c. Quality measures of postprocessing Model fitting (e.g. CRLB, goodness of fit, SD of residual) |  |
| d. Sample Spectrum | Figures 1 and 2 |

**Table S2:** FWHM values (in Hz) reported by LCModel across T_E_s and participants.

| **Participant #** | **T_E_ = 23 ms** | **T_E_ = 68 ms** | **T_E_ = 97 ms** | **T_E_ = 110 ms** |
| --- | --- | --- | --- | --- |
| **1** | 4.4 | 4.4 | 5.2 | 5.2 |
| **2** | 3.0 | 3.0 | 2.2 | 2.2 |
| **3** | 3.7 | 3.7 | 3.7 | 3.7 |
| **4** | 3.7 | 3.7 | 3.7 | 3.7 |
| **5** | 3.7 | 3.7 | 3.7 | 3.0 |
| **6** | 5.2 | 3.7 | 4.4 | 5.2 |
| **7** | 3.0 | 3.0 | 3.0 | 3.0 |
| **8** | 5.2 | 5.2 | 4.4 | 4.4 |
| **9** | 3.0 | 3.0 | 3.0 | 3.0 |
| **10** | 3.7 | 3.7 | 2.2 | 2.2 |

**Table S3:** Mean ± SD and range of CRLB (%) across T_E_s for Asp and Cth for all participants.

|  | **Asp** |  | **Cth** |  |
| --- | --- | --- | --- | --- |
| **T_E_ / ms** | **Mean ± SD (%)** | **Range (%)** | **Mean ± SD (%)** | **Range (%)** |
| **23** | 35 ± 28 | 7 – 96 | 7 ± 5 | 3 – 22 |
| **68** | 520 ± 505 | 31 – 999 | 9 ± 3 | 6 – 16 |
| **97** | 253 ± 394 | 22 – 999 | 16 ± 12 | 7 – 50 |
| **110** | 397 ± 431 | 27 - 999 | 15 ± 7 | 6 - 29 |
